# Their Functions and Limitations

**Published:** 1919-02-15

**Authors:** 


### SANATORIA.

### Their Functions and Limitations.

When the tourist on the Nile reaches the squalid little town of Assiut, he is always taken by the dragoman to the suburban cemetery. No tombstones or tumuli are there, but multitudes of little white-washed houses built as abodes for the spirits of the departed. The city of the living is noisy and dirty, silent and dazzling is the city of the dead.

Had we to build such a necropolis for the spirits of the dead who have died in this war, what a great shining city it would be, with more houses than London and Paris combined! Yet, even now, the roll is incomplete. Day by day, battalion after battalion are marching along to the city of the dead. For it has been computed that in Great Britain alone two hundred thousand men have contracted consumption in the course of war work, and most of these are doomed men unless the nation will save them. Two hundred thousand men! Surely the graveyards have already had their fill! Shall we not try to rescue some of these from the hands of Death?

Consumption is a germ disease. That is the pity of it. A man without a leg is a tragic figure; a man without eyes makes sound eyes weep; but a man with a thousand million invisible germs gnawing at his lungs makes a comparatively poor appeal to the compassion of the unimaginative multitude.

Yet the germs are as real and as deadly as shrapnel bullets; their victim is as much to be pitied as a man on a crutch, and it is incumbent upon the nation to take all possible measures to save the great army of consumptives.

How can the consumptive be best helped? Undoubtedly, the best thing we can do for consumptives under present social conditions is to send them to sanatoria. Consumption is largely a disease of dirt, darkness, bad air, and poor food, and can be most effectually and economically combated by the cleanliness, sunlight, fresh air, and good food which sanatoria provide. Sanatoria, then, must be multiplied. Instead of building little white houses for the spirits of the dead, we must do honour to the dead by building big white houses to succour the bodies of their living brethren. That certainly must be done, but some words of warning are necessary.

It must be remembered—as has been too often quite forgotten—that sanatoria are merely temporary expedients and makeshifts, and that it is the duty of the nation not only to treat individuals suffering from tuberculosis, but to eradicate the disease altogether. Tuberculosis can be resisted and defeated by sanatoria; but it can be eradicated only when every home is a sanatorium. Only in the home will the tubercle bacillus ever be compelled to sign a treaty of unconditional surrender, and the provision of sanatoria must not distract public attention from the much more vital need—the provision of healthy homes.

Sanatoria, then, are merely temporary expedients, and in a few decades they will be as useless and obsolete as leper-houses, and though they must be built now, they should be built as cheaply as possible. The expenditure of great sums on the erection of massive stone sanatoria, warranted to endure for centuries, is a gross waste of money. We do not require palatial buildings and grand hotels for the treatment of tuberculosis; if a sanatorium keeps out rain and lets in plenty of air and sunlight that is all that is requisite, and the less money spent on bricks and mortar the more remains to be spent on bread and butter and other things essential for the patient.

It must be noted, further, that the usefulness of a sanatorium depends in great measure on a careful selection of cases suitable for treatment. Even if it were possible to treat *all* consumptives it would be a mistake to do so; for a considerable proportion of cases are incurable, and money spent in endeavouring to cure the incurable would be money wasted. The cases must be selected, and they must be selected by experts with practical experience of open-air treatment, and no outside influence must be allowed to have power to secure the admission of unsuitable cases, however deserving. Sanatoria must not be used merely to give comfort and nursing attention to dying cases. That means not only the waste of beds that might restore more hopeful cases to health and usefulness, but, in addition, advanced and hopeless cases require an undue amount of attention and are apt to depress all their fellow-patients. If advanced and hopeless cases are to receive sanatorium treatment they must receive it in special sanatoria set apart for them and run on special lines.

Further, we must recognise that no sanatorium can undertake to cure permanently even the most favourable cases. Even after tuberculosis is apparently quite arrested, any illness or strain or any transgression of the rules of healthy living is quite likely to result in a recurrence of the disease. Any sanatorium, then, which is to effect permanent benefit must have associated with it some kind of colony which, for a time, at least, will provide suitable work, healthy environment, and adequate medical supervision for discharged patients.

Finally it must be clearly understood that a sanatorium with selected patients, and with machinery for after-care, must still depend for its complete success on the personality of the sanatorium doctor. Much has been written about choice of a site or choice of a dietary, or choice of a climate for a sanatorium, but singularly little about the choice of a sanatorium doctor. An impression seems to be abroad that one doctor is as good as another, and that the chief thing in sanatorium treatment is the sanatorium.

No graver mistake than this could be made. The doctor and not the building is the important thing; and the sanatorium without an expert doctor is like a kitchen without an expert cook. The principles of sanatorium treatment are few and simple;

**Sanatoria**—(*continued*).

but this renders their consistent and persistent observance all the more difficult. The average patient has not much scientific imagination nor much medical knowledge; he has grown up in the belief that the remedies for all his diseases are to be found in little bottles and boxes, and when he finds that treatment consists mainly of fresh air, simple food, and a regulated life he is apt to consider it no treatment at all, and to resent the discipline exercised over him. Yet it is just on a believing, punctilious observance of a few simple principles that cure depends.

The doctor with the personality to inspire confidence and to command obedience is not easily found, but is well worth finding. I have no hesitation in saying that the difference between a suitable and unsuitable doctor is, for 5 per cent. of the cases, the difference between life and death. Nor is a sanatorium merely an institution for diagnosis, prognosis, and treatment; it is a great centre of education and propaganda. The patients must not only be persuaded to obey irksome rules while in residence, but they must also be so convinced of the efficacy and value of the rules as to follow them and preach them after discharge. If a patient, after leaving a sanatorium, is not an evangelist of the doctrine of the open window, if he goes home and tolerates a foul atmosphere to please his wife, if he spits at large, then the sanatorium has fulfilled only half its hygienic function and the sanatorium doctor has performed only half his duty.

The ideal sanatorium is, in fact, not the sanatorium with the ideal construction; but the sanatorium with the ideal doctor.

